# Common genetic variants of the ion channel transient receptor potential membrane melastatin 6 and 7 (*TRPM6 *and *TRPM7*), magnesium intake, and risk of type 2 diabetes in women

**DOI:** 10.1186/1471-2350-10-4

**Published:** 2009-01-17

**Authors:** Yiqing Song, Yi-Hsiang Hsu, Tianhua Niu, JoAnn E Manson, Julie E Buring, Simin Liu

**Affiliations:** 1 Division of Preventive Medicine, Department of Medicine, Brigham & Women's Hospital, Harvard Medical School, Boston, MA, USA; 2 Institute for Aging Research, Hebrew Senior Life, Harvard Medical School, Boston, MA, USA; 3 Molecular and Integrative Physiological Science Program, Department of Environmental Health, Harvard School of Public Health, Boston, MA, USA; 4 Department of Epidemiology, Harvard School of Public Health, Boston, MA, USA; 5 Department of Ambulatory Care and Prevention, Harvard Medical School, Boston, MA, USA; 6 Department of Epidemiology, UCLA School of Public Health, Los Angeles, CA, USA; 7 Department of Medicine, David Geffen School of Medicine, University of California, Los Angeles, CA, USA

## Abstract

**Background:**

Ion channel transient receptor potential membrane melastatin 6 and 7 (TRPM6 and TRPM7) play a central role in magnesium homeostasis, which is critical for maintaining glucose and insulin metabolism. However, it is unclear whether common genetic variation in *TRPM6 *and *TRPM7 *contributes to risk of type 2 diabetes.

**Methods:**

We conducted a nested case-control study in the Women's Health Study. During a median of 10 years of follow-up, 359 incident diabetes cases were diagnosed and matched by age and ethnicity with 359 controls. We analyzed 20 haplotype-tagging single nucleotide polymorphisms (SNPs) in *TRPM6 *and 5 common SNPs in *TRPM7 *for their association with diabetes risk.

**Results:**

Overall, there was no robust and significant association between any single SNP and diabetes risk. Neither was there any evidence of association between common *TRPM6 *and *TRPM7 *haplotypes and diabetes risk. Our haplotype analyses suggested a significant risk of type 2 diabetes among carriers of both the rare alleles from two non-synomous SNPs in *TRPM6 *(Val1393Ile in exon 26 [rs3750425] and Lys1584Glu in exon 27 [rs2274924]) when their magnesium intake was lower than 250 mg per day. Compared with non-carriers, women who were carriers of the haplotype 1393Ile-1584Glu had an increased risk of type 2 diabetes (OR, 4.92, 95% CI, 1.05–23.0) only when they had low magnesium intake (<250 mg/day).

**Conclusion:**

Our results provide suggestive evidence that two common non-synonymous *TRPM6 *coding region variants, Ile1393Val and Lys1584Glu polymorphisms, might confer susceptibility to type 2 diabetes in women with low magnesium intake. Further replication in large-scale studies is warranted.

## Background

Magnesium is an essential mineral derived primarily from dietary sources, including whole-grains, green leafy vegetables, legumes, and nuts [1]. Adequate magnesium intake is critical in maintaining magnesium balance and normal magnesium-dependent cellular reactions in the human body. Magnesium deficiency has been correlated with a cluster of metabolic abnormalities and associated chronic diseases such as oxidative stress, systemic inflammation, endothelial dysfunction, insulin resistance, hypertension, type 2 diabetes, and coronary heart disease [2-6]. Although the precise mechanisms underlying magnesium metabolism are unclear, magnesium homeostasis in the human body seems to be tightly regulated via the balance between intestinal absorption and renal excretion. Recent studies suggest that transient receptor potential membrane melastatin 6 and 7 (TRPM6 and TRPM7), two members of the "transient receptor potential" (TRP) family of cation channels, may play a central role in the regulation of magnesium homeostasis [7-10].

The *TRPM6 *gene, located on chromosome 9q21.13, comprises 39 exons spanning 163 kb of genomic sequence and encodes 2022 amino acids [11]. TRPM6 is thought to be an ion channel subunit primarily expressed in intestinal epithelia and kidney tubules that may play an important role in intestinal and renal magnesium handling [12,13]. Several loss-of-function mutations in *TRPM6 *have been identified among patients with autosomal-recessive familial hypomagnesemia with secondary hypocalcemia [12-14]. *TRPM7*, located on chromosome 15q21, spans 127 kb of DNA sequence and encodes a protein of 1865 amino acids. TRPM7 is ubiquitously expressed in various tissues or cell lines [8,9] and may be part of a magnesium sensing and/or uptake mechanism underlying cellular magnesium homeostasis [8,9]. Although *TRPM6 *and *TRPM7 *share 52% homology [15], their biological functions are not redundant, and they can form functional heterometic channels with different permeability [16]. Thus, these two genes are candidate genes for evaluating the genetic basis of hypomagnesemia-related metabolic traits such as the common form of type 2 diabetes. To date, no study has assessed common genetic variation in these two genes or their contribution to susceptibility to chronic metabolic disorders highly correlated with hypomagenesemia. Furthermore, a magnesium-deficient diet was shown to upregulate *TRPM6 *mRNA expression in mice [17]. Low serum magnesium levels caused by *TRPM6 *mutations among HSH patients can be ameliorated by oral supplementation of high doses of magnesium [18]. Thus, it is important to further examine in human population data whether magnesium intake modifies any common genetic effects of *TRPM6 *and *TRPM7 *on diabetes risk.

We therefore conducted a prospective nested case-control study among postmenopausal women in the Women's Health Study to investigate the association between common variations of the *TRPM6 *and *TRPM7 *genes and the risk of type 2 diabetes. We specifically tested the following hypotheses: (1) common genetic variants in *TRPM6 *and *TRPM7 *are associated with risk of developing type 2 diabetes because of their critical functions on maintaining magnesium homeostasis; and (2) The genetic association, if any, is more pronounced among women with inadequate magnesium intake.

## Methods

### Study population

The Women's Health Study, begun in 1993, is a randomized, double-blind, placebo-controlled, 2 × 2 factorial trial of low-dose aspirin and vitamin E for the primary prevention of CVD and cancer among 39,876 US female health professionals aged 45 years and older free of cancer (other than nonmelanoma skin cancer), and cardiovascular disease [19,20] at baseline. Of the 39,876 participants, 28,345 (71%) provided baseline blood samples. Of these 28,345, we restricted our study to 12,304 women free of diabetes at baseline who were not currently receiving hormone replacement therapy and were postmenopausal at the time of blood collection. By February 2005 with 10 years of follow up, 366 of these initially healthy women had developed incident diabetes. Using the principle of risk-set sampling to randomly select controls from the cohort person-time [21], controls providing baseline blood samples were matched in 1:1 ratio to cases by age (within 1 year), duration of follow up (within 1 month), race, and fasting status at time of blood draw (72% provided fasting blood, defined as ≥ 10 hrs since last meal). Based on these criteria, a total of 359 cases and 359 controls were selected. This study was conducted according to the ethical guidelines of Brigham and Women's Hospital.

### Data collection

On the baseline questionnaire, we collected detailed information on demographics, medical history, and lifestyle factors. In the enrollment questionnaire, participants provided information on lifestyle exposures including cigarette smoking status (never, past, current), aerobic physical activity (never, <1/week, 1/week, 2–3 times/week, 4–6 times/week, daily), total intakes of beer, wine, and liquor (g/day), use of aspirin before the trial (yes, no), use of multivitamin (yes, no), and use of postmenopausal hormone therapy (never, past, current), Moreover, participants reported their medical history characteristics such as age, height, weight, history of type 2 diabetes, and family history of diabetes in first-degree relatives.

As described previously, a semiquantitative food-frequency questionnaire (SFFQ) at baseline was used to assess dietary nutrient intake [22]. Each nutrient was adjusted for total energy using the residual method [23]. In populations of nurses and health professionals, this SFFQ has demonstrated reasonably good validity as a measure of long-term average dietary intakes [24]. The Pearson correlation coefficient between magnesium intake assessed by SFFQ and 2 weeks of diet records was 0.76 [25]. The use of multivitamin supplements was taken into account to assess intake of supplemental magnesium. Total magnesium represents the sum of magnesium intake from both dietary and supplemental sources.

### Ascertainment of diabetes

After excluding prevalent cases at baseline, all eligible participants were asked annually whether and when they had been diagnosed with diabetes since completing the previous questionnaire. Women self-reporting incident diabetes in annual follow-up were mailed supplementary questionnaires to confirm diabetes symptoms, diagnostic tests, and treatments. Diabetes was confirmed according to the American Diabetes Association (ADA) criteria of 1997 [26]. Previous WHS diabetes validation via physician-led telephone interviews and self-administered supplementary questionnaires both yielded positive predictive values >91%, and confirmation of diabetes via combined supplementary questionnaire and medical records was 99% [27,28].

### SNP selection and genotyping

We initially surveyed common SNPs with minor allele frequency (MAF) ≥ 5% from the public accessible database, the National Center for Biotechnology Information database SNP (NCBI dbSNP) supplemented by the CEU HapMap database of the International HapMap project. Our goal was to select a minimal set of common SNPs (MAF ≥ 5%) to capture the common genetic variability across the genomic regions of the *TRPM6 *(223 kb) and of the *TRPM7 *(187 kb) genes, including their corresponding 30 kb 5' upstream and 30 kb 3' downstream regions. SNPs were selected based on the following criteria: 1) Functionality priority: non-synonymous coding SNPs, splicing-site SNPs, and promoter SNPs were kept; and 2) minor allele frequency (MAF) ≥ 5% in at least one Caucasian population [29]. In total, a set of 25 SNPs (20 for *TRPM6 *and 5 for *TRPM7*) were chosen and genotyped in the entire case-control sample (Table 1).

**Table 1:** The location, minor allele frequency, and crude association analyses of 25 SNPs spanning both *TRPM6 *and *TRPM7 *loci

			**Minor allele frequency (MAF)**		**P values for crude association^b^**		
			
**SNP**	**Alleles** **(major/minor)**	**Location^a^**	**Cases**	**Controls**	**Genotypic recessive**	**Genotypic additive**	**Genotypic dominant**
rs4745363 (46)	T>A	Intron 1	0.46	0.42	0.08	0.18	0.59
rs10869447 (45)	A>G	Intron 3	0.39	0.35	0.05	0.25	0.79
rs11144108 (44)	T>G	Intron 6	0.36	0.35	0.20	0.67	0.77
rs7867868 (43)	C>T	Intron 6	0.33	0.32	0.56	0.75	0.41
rs7045949 ^c^(42)	C>T (in only European whites)	Intron 7	0.41	0.42	0.91	0.76	0.59
rs1012710 (41)	T>C	Intron 12	0.30	0.30	0.93	0.97	0.91
rs10512038 (40)	T>C	Intron 14	0.25	0.28	0.83	0.28	0.21
rs7859201^d^(39)	A>C (Leu708Leu)	Exon 17	0.39	0.43	0.32	0.11	0.13
rs3858116 (38)	G>A	Intron 18	0.38	0.40	0.78	0.46	0.39
rs2151424 (37)	C>T	Intron 19	0.26	0.28	0.13	0.37	0.74
rs2151423 (36)	A>G (whites only)	Intron 19	0.45	0.46	0.43	0.91	0.60
rs6560408 (35)	C>T	Intron 23	0.39	0.40	0.90	0.74	0.56
rs3750425^e^(34)	G>A (Val1393Ile)	Exon 26	0.097	0.095	0.29	0.86	0.89
rs2274924^f^(33)	A>G (Lys1584Glu)	Exon 27	0.18	0.18	0.42	0.79	0.51
rs2769195 (32)	T>C	Intron 27	0.44	0.42	0.03	0.33	0.69
rs1327824 (31)	G>C	Intron 31	0.09	0.11	0.50	0.46	0.53
rs875034 (30)	A>G	Intron 33	0.42	0.42	0.91	0.84	0.83
rs944857 (29)^g^	T>C	Intron 36	0.02	0.009	--	0.07	0.07
rs539079 (28)	A>G	Intron 36	0.10	0.09	0.67	0.91	0.99
rs514348 (27)	T>A	Intron 38	0.41	0.40	0.09	0.80	0.35

***TRPM7***							
rs8042919^h^(51)	G>A (Thr1482Ile)	Exon 28	0.10	0.12	0.27	0.19	0.27
rs3109881(50)	G>A	3'UTR	0.36	0.41	0.15	0.04	0.07
rs10519279 (49)	G>C	3'UTR	0.16	0.19	0.40	0.19	0.22
rs3131597 (48)	C>T	3'UTR	0.41	0.46	0.10	0.13	0.36
rs3098198 (47)	A>G	3'UTR	0.47	0.41	0.13	0.03	0.03

a: The location was based on the contiguous position (reference NT_023935.17 for *TRPM6 *and NT_010194.16 for *TRPM7*);

b: Adjusted for two matching variables (age and race).

c: Exon-intron boundary region;

d: Synonymous polymorphism (Leu708Leu)

e: Non-synonymous polymorphism in *TRPM6*: Protein residue: Val1393 Ile

f: Non-synonymous polymorphism in *TRPM6*: Protein residue: Lys1584Glu

g: No homozygosity observed for rare allele A;

h: Non-synonymous polymorphism in *TRPM7*: Protein residue: Ile482Thr

DNA was extracted from the buffy coat fraction of centrifuged blood using a QIAmp blood kit (Qiagen, Chatsworth, CA) at the Dana Farber/Harvard Cancer Center High Throughput Genotyping Core (David J. Hunter, MD, Director). DNA samples were genotyped using Taqman single nucleotide polymorphism allelic discrimination by means of an ABI 7900 HT (Applied Biosystems, Foster City, CA). The primers and probes were custom-designed by the ABI Taqman system (PE Biosystems, Foster City, CA). Following PCR amplification, end-point fluorescence was read with the Applied Biosystems Primer 7900 HT instrument and genotypes were assigned using SDS2.2.2 Allelic Discrimination Software (Applied Biosystems, Foster City, CA) by a technician blinded to sample identification numbers. Replicate quality control samples (10%) were included and genotyped with >99% concordance.

### Statistical analyses

Distributions or proportions of baseline characteristics of study subjects were examined according to case-control status. Student's t-test and chi-square test were used for comparisons of means and proportions. We assessed each SNP for the Hardy-Weinberg equilibrium (HWE) test using the chi-square test.

Pairwise LD between SNPs was assessed using Lewontin's D' statistic and the squared correlation statistic *r*^2^. The Haploview program was used to calculate the LD coefficient and define haplotype blocks [30,31]. For each SNP, we tested for allelic association with diabetes risk under dominant, recessive, and additive models. We used unconditional logistic regression to estimate odds ratios (ORs) and 95% confidence intervals (CIs) for each SNP with diabetes risk for each genetic model, respectively. We made adjustments for multiple confounding variables including matching factors (age and ethnicity), BMI (continuous), smoking (current, former, and never), alcohol use (rarely/never drinkers, 1–3 drinks/month, 1–6 drinks/week, and 1 or more drinks/day), exercise (rarely/never, 1, 2–3, 4–6 and ≥ 7 times/week), and family history of diabetes (yes, no).

Haplotypes inferred from all SNPs and all haplotypes within each LD block were tested for association with type 2 diabetes. We estimated haplotype frequency from phase-unknown genotype data using the expectation-maximization (EM) algorithm [32]. For each individual and each haplotype, *h*, the haplotype dosage estimate (i.e. an estimate of the number of copies of haplotype *h*) was computed using the individual's genotype data and haplotype frequency estimates obtained from the combined (cases+controls) data set [32,33]. Only haplotypes with estimated frequencies ≥ 1% in the combined cases and controls were included for analyses. We first performed global likelihood ratio tests to examine whether the frequency distributions of the common haplotypes differed between cases and controls by comparing a model with additive effects on the log odds scale for each common haplotype (using the most common halpotype as the reference) to the intercept-only model. As compared to the corresponding non-carriers of each haplotype, we also calculated haplotype-specific ORs and 95% CIs for carriers of each specific haplotype from logistic regression analyses.

A sliding window (window width = 2) haplotype-based analysis was performed to identify a "sub-haplotype" that would be most significantly associated with the disease outcome. The omnibus likelihood ratio test was used to test the association significance in the total samples, and in subsamples stratified by levels of magnesium intake. A -log_10_p > 2.88 (p < 0.0013) was used as the global significance threshold by Bonferroni correction for 19 window frames.

Stratified analyses were conducted to examine whether the association between the *TRPM6 *and *TRPM7 *genotypes and risk of type 2 diabetes was modified by magnesium intake. The cutpoints for magnesium levels were prespecified by those for quintiles among controls. Interactions between the genotype/haplotype and magnesium intake were assessed by comparison of regression models with and without the product terms between the genotype and stratifying variables using a likelihood ratio test.

All reported P-values were 2-tailed, and statistical significance was defined at the α = 0.05 level. Statistical analyses were performed using SAS statistical package (version 9.0 for window; SAS Institute, Cary, NC). In addition, we performed bioinformatics analyses to address the potential functional relevance of three common coding SNPs in *TRPM6 *and *TRPM7*. Several algorithms including PMut [34], PolyPhen [35], and SIFT [36] were employed to predict the impact of these amino acid substitutions on protein structure and activity. The principles for these algorithms are sequence conservation over evolutionary time, the physical and chemical properties of the exchanged residues, and/or protein structural domain information. Different algorithms emphasize different aspects of assumptions, quantitative measures, and dynamic databases, but these algorithms are highly capable of predicting the impact of amino acid substitutions on function, consistent with results from biochemical analyses (>80% concordance rate). Polymorphisms within highly conserved regions are likely to be of greater functional significance than those within more diverged regions. Our cross-species comparison approach for *TRPM6 *and *TRPM7 *was based solely on the availability of relevant information from the NCBI Genbank database. To provide some indirect evidence for protein sequence changes among different species, cross-species comparison of the protein sequences was performed for two *TRPM6 *segments encompassing the two non-synonymous SNPs, V1393I and K1584E in human, chimpanzee, and mouse and a *TRPM7 *segment encompassing the non-synonymous SNP, T1482I in human, Sheep, mouse, and zebra fish. Protein sequence alignments were performed by use of ClustalX, version 1.81 [37].

## Results

Basic characteristics of the population by case-control status are shown in Table 2. Overall, diabetes cases had a higher prevalence of traditional diabetes risk factors at baseline than controls. The location, minor allele, and allele frequency for all 25 SNPs are shown in Table 1. Their observed genotype frequencies, except for rs7867868 (P = 0.03), were within HWE among the controls. We examined whether each of the SNPs chosen was significantly associated with type 2 diabetes under three genetic models (additive, recessive, and dominant models). As showed in Table 1, only one SNP in *TRPM6 *was significantly associated with diabetes risk in the recessive model (rs2769195, P = 0.03). Of the 5 *TRPM7 *SNPs, rs3109881 and rs3098198 showed significant associations with diabetes risk (rs3109881, additive P = 0.04; and rs3098198, additive *P *= 0.03 and dominant P = 0.03). These results were likely to be false positives, because they did not remain statistically significant after further adjustment for more covariates or multiple comparisons.

**Table 2:** Baseline characteristics of patients with type 2 diabetes and control participants.

**Characteristics^a^**	**Cases** n = 359	**Controls** n = 359	**P value^b^**
Age, years	60.3 ± 6.1	60.3 ± 6.1	--^c^
Race (Caucasian), %	92.5	92.5	--^c^
BMI, kg/m^2^	30.9 ± 6.1	26.0 ± 5.0	<0.0001
Cigarette smoking (%)			
Never	47.5	49.6	0.85
Past	38.0	36.8	
Current	14.5	13.7	
Physical activity, %			
Rarely/never	51.1	39.6	0.008
1 to 3 times per month	18.2	21.7	
> 1 time per week	30.7	38.7	
Alcohol consumption, %			
Rarely/never	60.7	47.1	0.0006
1 to 3 times per month	13.7	15.3	
>1 time per week	25.6	37.6	
Family history of diabetes (%)	48.5	24.0	<0.0001
Dietary factors			
Total calorie intake, kcal/d	1809 ± 592	1722 ± 554	0.05
Magnesium intake, mg/d ^d^	329 ± 70.5	348 ± 83.0	0.001

^a ^All data are mean ± SD unless indicated otherwise;

^b ^P value from chi-square and t tests;

^c ^Matching variables;

^d ^Magnesium intake was the total amount of magnesium from both food sources and multivitamin supplements and was energy-adjusted.

**Figure 1:**
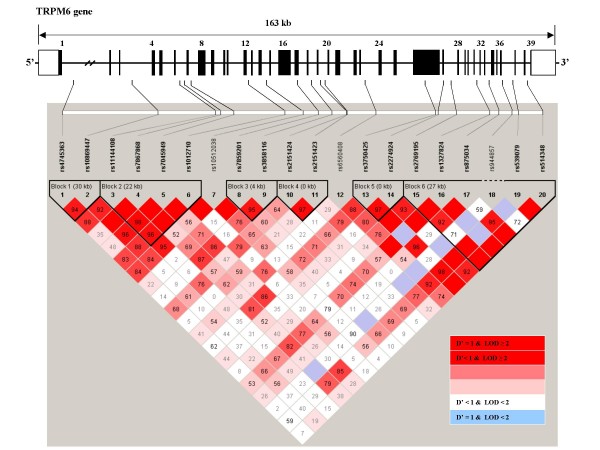
**Location and linkage disequilibrium (LD) map of 20 SNPs in *TRPM6***. The relative physical position of each SNP is given in the upper diagram. Exons are represented by solid bars; intronic and 5' and 3' regions are represented by solid lines. The dbSNP reference numbers are indicated below each SNP. The pairwise linkage disequilibrium (LD) coefficient D' for the control participants were calculated using Haploview. Each diamond for each SNP combination indicates the pairwise LD between all tSNPs, with red indicating strong LD (D' > 0.8) and a logarithm of odds score of >2.0. LD strength between the chosen SNPs is determined by the 90% confidence limits of D' statistic. Haplotype blocks were identified using Haploview.

**Figure 2:**
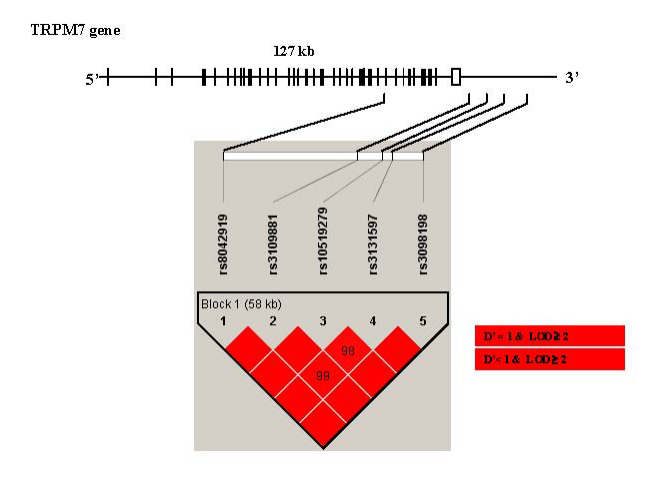
**Location and linkage disequilibrium (LD) map of 5 SNPs in *TRPM7 *using Haploview**. The relative physical position of each SNP is given in the upper diagram. Exons are represented by solid bars; intronic and 5' and 3' regions are represented by solid lines. Each diamond for each SNP combination indicates the pairwise LD between all tSNPs, with red indicating strong LD (D' > 0.8) and a logarithm of odds score of >2.0. LD strength between the chosen SNPs is determined by the 90% confidence limits of D' statistic.

The LD structure and haplotype blocks were shown for 20 SNPs in *TRPM6 *(Figure 1) and 5 SNPs in *TRPM7 *(Figure 2) among controls. The 20 SNPs in *TRPM6 *fall into six blocks with high LD and different sizes. Notably, the two non-synonymous SNPs (rs3750425 and rs2274924) in *TRPM6 *were 762 bp apart and yet not in complete LD (D' = 0.98; r^2 ^= 0.46). For *TRPM7*, rs8042919 (Ile1482Thr in exon 29) was in tight LD with all other SNPs in *TRPM7 *(D' = 1.00; r^2 ^= 0.10–0.59).

Haplotype analyses of the 20 SNPs spanning the *TRPM6 *region did not reveal any significant associations. There was no evidence for haplotypic associations with all derived haplotypes based on all 20 *TRPM6 *SNPs and 5 *TRPM7 *SNPs. Haplotype analysis in each of these blocks did not provide evidence of statistically significant associations with diabetes risk (data not shown). Overall, a global test for differences in risk according to common haplotypes (≥ 1%) inferred from 20 SNPs over *TRPM6 *was not statistically significant (P = 0.31). There was no significant association with haplotypes in any single block: p values for global testing of haplotype associations were 0.69 for block 1, 0.96 for block 2, 0.12 for block 3, 0.92 for block 4, 0.85 for block 5, and 0.33 for block 6. Neither was there any evidence of association between common *TRPM7 *haplotypes and diabetes risk (P for global test = 0.43).

**Figure 3:**
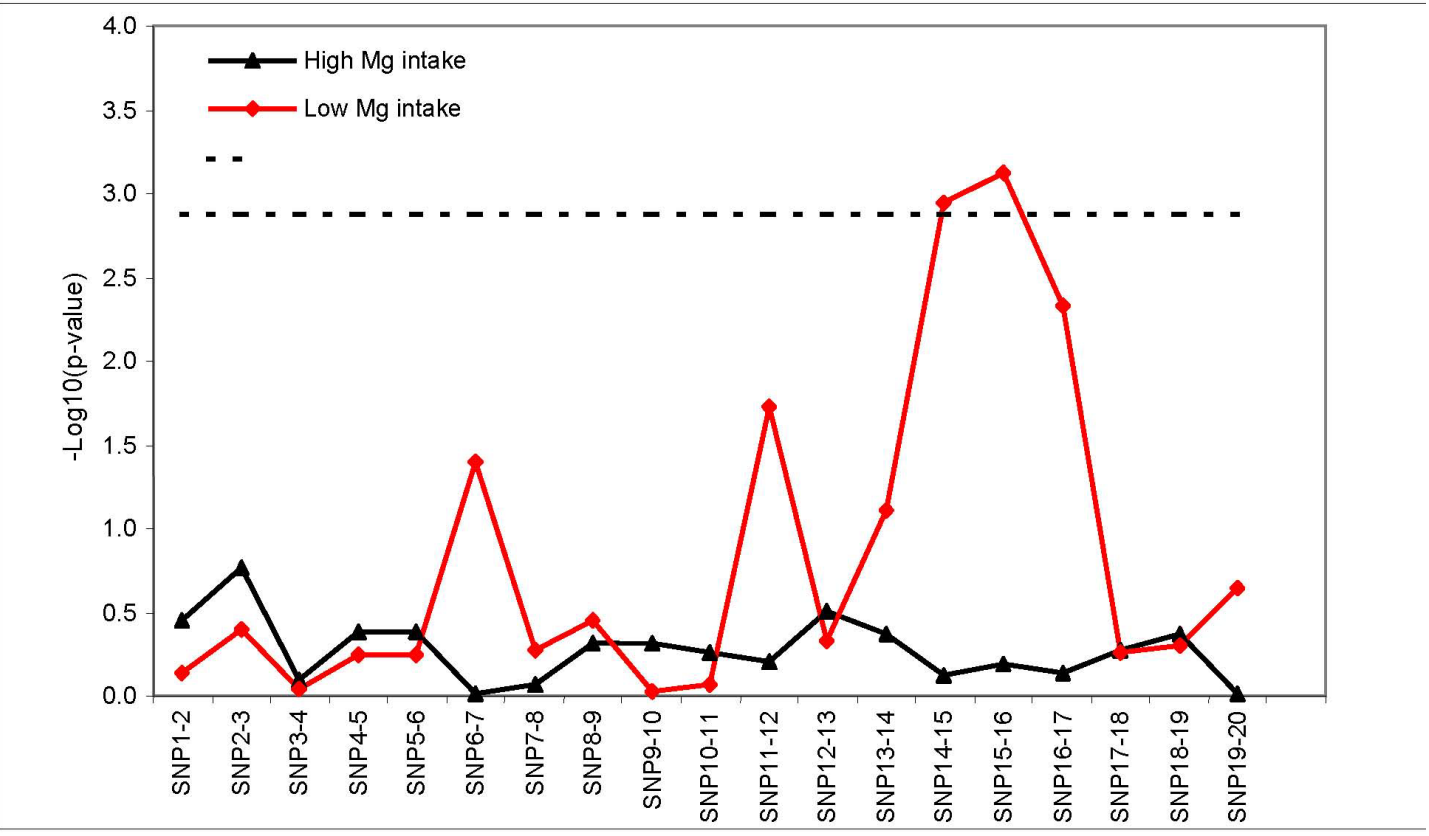
**Sliding window analysis for haplotype-disease associations**. Haplotype was reconstructed by 2-SNP sets. The omnibus test was used for the *TRPM6 *haplotype-type 2 diabetes associations for each sliding window set. Empirical p-value was based on permutation test. The dashed line represents a -log_10_P value of 2.88 (corresponding to P = 0.0013), which was used as the global significance threshold by Bonferroni correction for 19 window frames.

We conducted a sliding window (window width = 2) haplotype-based analyses to examine the genetic associations of *TRPM6 *with risk of type 2 diabetes. There were a total of 19 window frames for the 20 SNPs. Overall, there were no significant associations for any 2-SNP "sub-haplotype" in any case-control samples (data not shown). When we stratified all cases and controls by magnesium intake, only one 2-tSNP "sub-haplotype", rs3750425-rs2274924 (*χ*^2 ^= *24.14, d.f. = 7, p = 0.0011)*, reached global significance (i.e. -log_10 _p > 2.88) for women with magnesium intake in the lowest 20% (<250 mg/day) (Figure 3). These two SNPs were both non-synonymous and in a tight LD block, with the frequency of carriers for both rare alleles of these two SNPs (the *A *allele of rs3750425 and the *G *allele of rs2274924) representing 9.3% in all the control samples. As shown in Table 3, there were no significant associations between any individual non-synonymous SNP and diabetes (under dominant models), among women with either low or high magnesium intake. Only among women with low magnesium intake was the haplotype based on 2SNPs in *TRPM6 *significantly associated with diabetes risk, although statistical power in our subgroup analyses became weak. Among women with low magnesium intake, ORs and 95% CIs in a comparison with carriers of both common alleles (G-A) were 4.92 (1.05–23) for the haplotype A-G and 0.56 (0.09–3.52) for the haplotype G-G. Adjustment for more potential confounders did no materially change the results.

**Table 3:** Association between three non-synonymous polymorphisms in *TRPM6 *and *TRPM7 *and the risk of type 2 diabetes among women stratified by magnesium intake

			**Magnesium Intake**			
			
	**Number**		**Low** **(<250 mg/day)**		**High** **(≥ 250 mg/day)**	
	
**Genotype^a^**	Cases	Controls	Age, race, and BMI-adjusted OR (95% CI)	Multivariate-adjusted OR (95% CI)^b^	Age, race, and BMI-adjusted OR (95% CI)	Multivariate-adjusted OR (95% CI)^b^
***TRPM6***						
rs3750425 (Val1393Ile)						
GG	279	287	1.00	1.00	1.00	1.00
GA/AA	60	64	4.32 (0.98–19.1)	5.29 (0.96–29.1)	0.90 (0.57–1.44)	0.80 (0.49–1.31)
rs2274924 (Lys1584Glu)						
AA	239	230	1.00	1.00	1.00	1.00
AG/GG	106	114	2.19 (0.68–7.11)	2.16 (0.58–8.07)	0.89 (0.61–1.31)	0.88 (0.59–1.31)
Haplotype^c^						
0–0 (G-A)	82.4%	81.5%	1.00	1.00	1.00	1.00
1–1 (A-G)	9.86%	9.30%	4.92 (1.05–23.0)	5.80 (1.01–33.5)	0.95 (0.62–1.44)	0.87 (0.56–1.35)
0–1 (G-G)	7.72%	9.03%	0.56 (0.09–3.52)	0.44 (0.06–3.26)	0.94 (0.61–1.46)	1.03 (0.66–1.63)
***TRPM7***						
rs8042919 (Thr1482Ile)						
GG	278	272	1.00	1.00	1.00	1.00
AG/AA	63	76	2.48 (0.48–12.7)	3.60 (0.56–23.0)	0.93 (0.60–1.44)	0.97 (0.61–1.52)

a: Only haplotypes with frequency > 1% are reported

b: Adjusted for matching variables (age and race), BMI, smoking, alcohol use, exercise, and family history of diabetes.

c: Haplotypes (rs3750425/rs2274924) were inferred from phase-unknown genotype data using the expectation-maximization algorithm.

**Figure 4:**
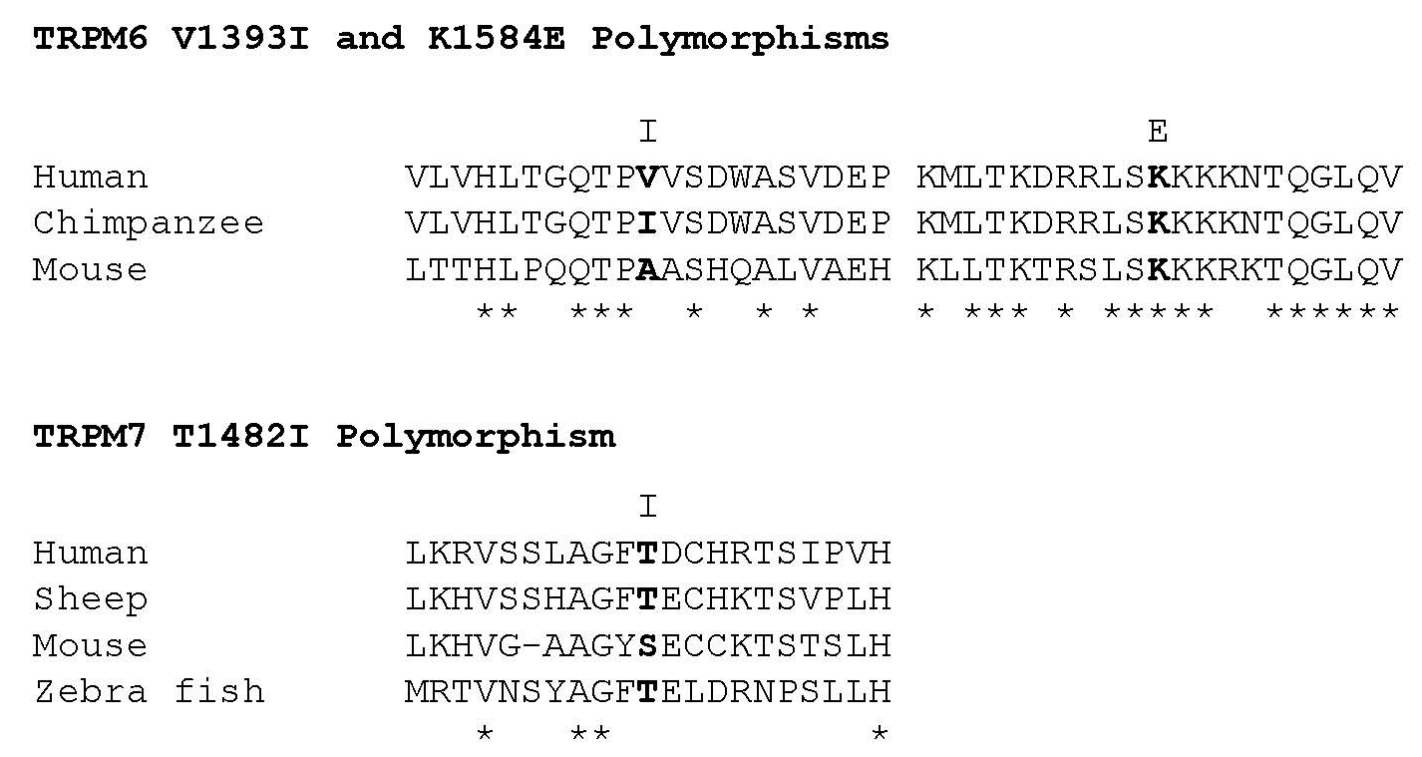
**Cross-species comparison of the protein sequences of (A) two *TRPM6 *segments encompassing the two non-synonymous SNPs, V1393I and K1584E (21 amino acids each, centered at the non-synonymous SNP) in human (GenBank accession number **Q9BX84), **chimpanzee (GenBank accession number **XP_528327), **and mouse (GenBank accession number **Q8CIR4); **and (B) a *TRPM7 *segment encompassing the non-synonymous SNP, T1482I (21 amino acids, centered at the non-synonymous SNP) in human (GenBank accession number **Q96QT4), **Sheep (GenBank accession number **ABP35923), **mouse (GenBank accession number **Q923J1), **and zebra fish (GenBank accession number **NP_001025232). The asterisks denote amino acid residues conserved across all the species that were compared.

In addition, our bioinformatics analyses provided some indirect evidence for prediction of the effects of an amino acid substitution SNP on protein structure and function (Figure 4 and Table 4). One non-synonymous SNP (rs2274924; K1584E) in *TRPM6 *seemed to be evolutionarily conservative and were predicted to be neutral using PMut prediction algorithm; however, measures of Polyphen and SIFT scores predicted that rs3750425 (V1393I) in *TRPM6 *and rs8042919 (T1482I) in *TRPM7 *might have a negative impact on protein activity. It should be noted that these algorithms are unable to predict the impact of multiple amino acid substitutions in a subunit on the activity of the protein. Thus, we cannot completely rule out the possibility that haplotypes based on multiple coding SNPs are functionally important.

**Table 4:** Computational predictions of the functional significances of 3 non-synonymous SNPs in the *TRPM6 *and *TRPM7 *genes

**rs#**	**SNP**	**Functional Prediction Algorithm**		
		
		**PMut Score** **[Reliability index]^a^**	**PolyPhen score^b^**	**SIFT Score^c^**
rs3750425	Val1393Ile (*TRPM6*)	0.0250 [9] (Neutral)	1.026 (Borderline)	0.04 (Intolerant)
rs2274924	Lys1584Glu (*TRPM6*)	0.2565 [4] (Neutral)	0.256 (Benign)	<0.001 (Intolerant)
rs8042919	Thr1482Ile (*TRPM7*)	0.7987 [5] (Pathological)	1.079 (Borderline)	0.03 (Intolerant)

a. The range of the PMut prediction score is from 0 to 1; a score over 0.5 suggests an effect on protein function and a score <0.5 indicates that the substitution is likely neutral. The range of the reliability index is from 0 to 9; the higher the number, the more reliable is the prediction.

b. PolyPhen Scores: 1.49–1.25 are designated as "Potentially damaging to function"; 1.24–1.00, "Borderline"; and <1.00, "Benign".

c. The SIFT scores are limited to the range of 0.0 to 1.0. Variants are classified as "Intolerant" (0.00–0.05), "Potentially intolerant" (0.051–0.10), "Borderline" (0.101–0.20), and "Tolerant" (0.201–1.00).

## Discussion

Overall, we observed no significant associations between the *TRPM6 *and *TRPM7 *genetic variants, individually or jointly, with risk of type 2 diabetes in women. However, women who were homozygous carriers of both the rare alleles of two non-synonymous SNPs in *TRPM6 *and had low magnesium intake had a significantly higher risk of type 2 diabetes, although these findings may have been due to chance and further large-scale studies are needed to confirm these subgroup analyses.

To the best of our knowledge, this is the first population study to comprehensively investigate *TRPM6 *and *TRPM7 *as potential candidate genes for the common form of type 2 diabetes. On the basis of the publicly accessible dataset, we focused on common SNPs including intronic SNPs, synonymous SNPs, and non-synonymous SNPs to characterize genetic variation spanning the *TRPM6 *gene, including at least 30 kb upstream and downstream of the coding regions. There may be some unknown causal SNPs including splicing SNPs and SNPs in the promoter region of *TRPM6 *that likely regulate gene expression. However, we could not identify regulatory SNPs without functional data and did not find any common SNP in the promoter region. Although we did not sequence genomic regions in *TRPM6 *in our study samples, empirical data have demonstrated that tSNPs selected from the HapMap population samples can effectively capture common variation and provide good power to detect a modest association in many other independent samples [38]. We cannot completely rule out the possibility of rare but functional variants (with an allele frequency <5%). Nevertheless, such rare alleles may not be major contributors to the most common late-onset form of type 2 diabetes with a high prevalence and incidence in the general population.

Our most significant statistical finding focused on two coding SNPs in *TRPM6 *among women with insufficient magnesium intake (at the lowest 20 percentile) (i.e. <250 mg/d), close to three quarters of the RDA for adult women. Among women, only those with low magnesium intake were found to have increased risk of type 2 diabetes. In our study population, approximately 33% are heterozygous for the rare *G *allele of rs2274924 and 18% heterozygous for the allele *A *of rs3750425. Both SNPs are also very common in populations with various genetic backgrounds; the HapMap population frequencies are 0.07–0.21 for the rs3750425 *A *allele (1393 Ile) and 0.15–0.36 for the rs2274924 *G *allele (1584Glu) in four ethnic groups. Two changed amino acids are located between the coiled region and kinase near the C-terminal [14]. Although such two amino acid substitutions in this region are unlikely responsible for direct regulation of the TRPM6 channel trans-membrane structures and kinase activity, they may alter protein conformation and thus reduce TRPM6 channel activity. Our bioinformatics approaches indicated that these coding SNPs in highly conserved region might be functionally significant, although further functional analysis should be warranted. There is also the possibility that these SNPs are in high LD with untyped SNPs that directly affect channel activity. Alternatively, two amino acid substitutions may be necessary to influence channel protein structure and function. Individuals with both rare alleles, whose TRPM6 channel function is deficient in magnesium absorption, may be more prone to magnesium deficiency through low intake and in turn to an increased risk of diabetes. If our finding is replicated in future studies, it will suggest that common genetic variation in the *TRPM6 *locus known to harbor severe mutations causing monogenic magnesium deficiency confers a modest susceptibility to the risk of type 2 diabetes in a small subgroup of the general population.

We found no significant evidence for an association between *TRPM7 *genetic variants and diabetes. Given the limited number of genotyped SNPs (n = 5) across *TRPM7 *(128 kb) from the reference panel (HapMap database), it is likely that they are not sufficient to capture the vast majority of the genetic variability of *TRPM7*. Biologically, *TRPM7 *is ubiquitously expressed and its constitutive activation is required for cellular survival. Animal studies showed that dietary magnesium restriction did not alter *TRPM7 *mRNA expression in mouse kidney and colon [17]. We therefore hypothesize that *TRPM7*, as a housekeeping gene regulating cellular magnesium metabolism, may truly have limited genetic variability.

The precise mechanisms for the regulatory role of *TRPM6 *and *TRPM7 *in magnesium homeostasis remain largely undefined. TRPM6 is a magnesium-permeable channel protein localized to the apical domain of the distal convoluted tubule and the brush-border membrane of the absorptive cells in intestine and plays an important role in active magnesium handling in the small intestine and kidney. Rare mutations in *TRPM6*, either incomplete or complete loss-of-function, have been associated with cases of autosomal-recessive hypomagnesemia with secondary hypocalcemia [12,13]. Clinical data show that sufficient magnesium intake can partially compensate for the severe magnesium deficiency caused by genetic defect in the *TRPM6 *gene [13]. It has been hypothesized that intestinal magnesium absorption occurs via two different pathways: an active transcellular transport and a passive paracellular passive transport [13]. High magnesium concentrations in intestinal tracts may overcome the genetic defects of magnesium absorption and independently increase magnesium absorption via the passive paracellular pathway. In contrast, when dietary magnesium intake is inadequate, the function of *TRPM6 *in active intestinal magnesium absorption and renal reabsorption become very important. Further well-designed functional studies are needed to investigate the molecular consequences of common genetic variability of *TRPM6 *and *TRPM7 *on mRNA expression, splicing, and degradation and metabolic profiles.

The strengths of our study include a prospective study design with up to 10 years of follow-up, careful collection of DNA samples and baseline variables, and an effective matching strategy used in a well-characterized study population. However, several limitations deserve further consideration. First, our study had relatively small sample size and did not have the power to assess modest genetic effects or interactions. The associations we identified could have been caused by chance due to statistical fluctuation; however, consistency with laboratory findings makes our results plausible. Second, population stratification may be a concern but unlikely to explain our findings, because our populations are racially homogeneous, with the majority of the participants being white (>92.5%). Third, we lacked stable and reliable measures of extracellular or intracellular magnesium status. Serum magnesium may be a good indicator for severe magnesium deficiency but is not sensitive to suboptimal magnesium status. Finally, findings in this cohort of women of predominantly European descent could not address population-specific genetic effects.

## Conclusion

Our results suggest that two common non-synonymous *TRPM6 *coding region variants might interact with magnesium intake in determining the risk of type 2 diabetes. However, our findings could be caused by chance due to multiple comparisons and insufficient statistical power; additional studies are needed to confirm these results and to further characterize the effects of genetic variation in *TRPM6 *on magnesium homeostasis.

## Competing interests

The authors declare that they have no competing interests.

## Authors' contributions

YS was responsible for the conception and design of the study. JEB and SL were responsible for acquisition of data. YS carried out the statistical analyses and drafted the manuscript. YHH and TN oversaw all the population genetics analyses and bioinformatics analyses and data interpretation. YHH, TN, JEM, JEB, and SL critically revised the manuscript for important intellectual content. YS, JEM, JEB, and SL obtained funding and provided administrative, technical, and material support. All authors read and approved the final manuscript.

## Pre-publication history

The pre-publication history for this paper can be accessed here:


